# A Unique Presentation of Immune Effector Cell-Associated Neurotoxicity Syndrome (ICANS) Presenting as Foot Drop in B-cell Acute Lymphoblastic Leukemia: A Case Report

**DOI:** 10.7759/cureus.88214

**Published:** 2025-07-18

**Authors:** Adelyn D Souza, Chinmay Patel, Karthick N Vijayaraghavan, Qi-An Wang, Amit Correa

**Affiliations:** 1 Department of Hematology, K. S. Hegde Medical Academy, Mangalore, IND; 2 Department of Hematology, Baroda Medical College, Maharaja Sayajirao University of Baroda, Gujarat, IND; 3 Department of Hematology, Stanley Medical College, Chennai, IND; 4 Department of Hematology, Chang Gung University School of Medicine, Taoyuan, TWN; 5 Department of Hematology, Lyndon B. Johnson Hospital, Houston, USA; 6 Department of Hematology, University of Texas Health Science Center, Houston, USA

**Keywords:** b-cell all (acute lymphoblastic leukemia), blinatumomab, cns relapse, foot drop, immune effector cell-associated neurotoxicity syndrome (icans)

## Abstract

Central nervous system (CNS) involvement in adult B-cell acute lymphoblastic leukemia (B-ALL) continues to pose treatment challenges, even with advancements in chemotherapy and targeted immunotherapy. Blinatumomab, a bispecific T-cell engager, has been shown to improve outcomes for patients with relapsed or refractory B-ALL; however, it can lead to immune-related neurotoxic effects, including immune effector cell-associated neurotoxicity syndrome (ICANS). We present an unusual case of a 38-year-old man with refractory B-ALL who experienced a parenchymal CNS relapse that manifested as foot drop during treatment with blinatumomab, likely as a result of ICANS. Initially, the patient achieved a remission with no minimal residual disease after undergoing R-HyperCVAD (rituximab, cyclophosphamide, vincristine, doxorubicin, and dexamethasone) and intrathecal chemotherapy. Unfortunately, he later relapsed with CNS involvement confirmed by imaging and biopsy. Following salvage therapy that included blinatumomab, he developed acute neurotoxic symptoms and peripheral neuropathy, which did not improve despite treatment. This case illustrates the complexities of CNS relapse in B-ALL and highlights a rare occurrence of neurotoxicity associated with blinatumomab, stressing the importance of awareness and prompt management of such complications.

## Introduction

Acute lymphoblastic leukemia (ALL) is a hematologic malignancy characterized by the proliferation of immature lymphoid cells in the bone marrow and peripheral blood. Among its subtypes, B-cell ALL (B-ALL) is the most prevalent in adults. Despite significant advancements in treatment, central nervous system (CNS) involvement remains a major clinical challenge, as leukemic infiltration into the CNS can lead to relapse even after achieving remission in the bone marrow and peripheral blood. At diagnosis, CNS involvement is observed in approximately 10% of adults with ALL [1]. The blood-brain barrier serves as a sanctuary site for leukemia, often shielding leukemic cells from systemic therapy [1].

The adoption of intrathecal chemotherapy as a prophylactic strategy has led to a significant decline in relapse rates. However, despite aggressive preventive measures, CNS recurrence remains an ongoing issue, with some studies reporting rates as high as 12.4% [2].

The incorporation of rituximab into a modified HyperCVAD regimen has demonstrated a 94% complete remission rate in patients with Philadelphia chromosome-negative (Ph−) B-ALL, highlighting its efficacy in improving treatment outcomes [3]. Additionally, the emergence of novel targeted therapies, such as monoclonal antibodies (e.g., rituximab, inotuzumab ozogamicin) and bispecific T-cell engagers (e.g., blinatumomab), has improved overall survival rates, but their precise role in CNS disease control continues to be explored. Blinatumomab has demonstrated notable efficacy in patients with relapsed or refractory B-ALL. However, its use is frequently linked to immune-related adverse effects, particularly cytokine release syndrome (CRS) in about 51% of patients and immune effector cell-associated neurotoxicity syndrome (ICANS) in about 26% of patients [4]. ICANS is an acute neurological complication caused by cytokine-driven inflammation, blood-brain barrier disruption, and immune cell infiltration into the CNS. It characteristically presents as altered mental status, language impairment, seizures, and, in severe cases, cerebral edema [4]. 

While peripheral neurotoxicity has been described with blinatumomab, most commonly as paresthesias, hypoesthesia, or tremor, mononeuropathies involving specific peripheral nerves such as the sciatic nerve remain unreported in clinical trials and post-marketing surveillance data [5]. Differentiating CNS relapse from ICANS is key, as the former presents with gradually evolving focal deficits or seizures and evidence of leukemic infiltration, while ICANS occurs acutely after immunotherapy, with diffuse encephalopathy, language disturbances, or seizures linked to systemic immune activation rather than direct CNS involvement [6].

This case report presents a unique instance of parenchymal CNS relapse in a patient with refractory B-ALL, with a rare complication of foot drop, which is potentially associated with blinatumomab-induced ICANS.

## Case presentation

A 38-year-old man presented to the emergency department (ED) on September 9, 2022, with complaints of chest pain and lower back pain for one day. He also reported symptoms of cough, recurrent fevers, malaise, and easy bruising for one month. On physical examination, multiple ecchymoses were noted on his left thigh, left lower extremity, and left upper arm.

Laboratory investigations revealed significant leukocytosis (99,800/µL) with 80% lymphoblasts, thrombocytopenia (21,000/uL), and a deranged coagulation profile: prothrombin time (PT) 15 seconds and partial thromboplastin time (PTT) 43.1 seconds. Peripheral blood flow cytometry revealed a marked increase in circulating B lymphoblasts (>75%) expressing CD10, CD19, CD20 (79%), CD22, CD38, and HLADR, suggestive of B-ALL (Table 1). Karyotyping demonstrated complex cytogenetic abnormalities, including deletions at 2p21 and 6q21q23, a derivative chromosome der(17)t(1;17)(q21;p11.2) in four cells, and a normal male karyotype (46 XY) in 16 cells. Fluorescence in situ hybridization (FISH) was negative for Philadelphia chromosome-like ALL. Cerebrospinal fluid (CSF) flow cytometry was positive for atypical cells at diagnosis. Computed tomography (CT) of the abdomen and pelvis revealed bilateral enhancement of the kidneys with multiple masses and mediastinal, periaortic, and retroperitoneal lymph node enlargement, indicating extramedullary disease, which may be seen in about 21% of patients at diagnosis [6].

**Table 1:** Laboratory investigations on initial presentation *Abnormal value based on institutional reference range

Test	Result	Normal range
Red blood cell count	4.33×10^6^/µL	4.2-5.9×10^6^/µL
Hemoglobin	10.5 g/dL*	14-17 g/dL
Hematocrit	31.9%*	41-51%
Mean corpuscular volume (MCV)	73.7 fL*	80-100 fL
Mean corpuscular hemoglobin (MCH)	24.2 pg*	28-32 pg
Mean corpuscular hemoglobin concentration (MCHC)	32.9 g/dL	32-36 g/dL
Platelet count	21,000/µL*	150,000-350,000/µL
Mean platelet volume	8.8 fL	7.2-11.7 fL
White blood cell count	99,800/µL*	4000-10000/µL
Differential count		
Neutrophils	4.0×10^3^/µL	1.5-8.0×10^3^/µL
Neutrophils (absolute)	4.53×10^3^/µL	1.5-8.0×10^3^/µL
Band	0	0
Lymphocytes	17.6×10^3^/µL*	1.0-4.0×10^3^/µL
Lymphocytes (absolute)	19.26×10^3^/µL*	1.0-4.0×10^3^/µL
Atypical lymphocyte	1.4×10^3^/µL*	0.12-0.48×10^3^/µL
Monocytes	6.0×10^3^/µL*	0.2-1.0×10^3^/µL
Eosinophils	0	0.0-0.5×10^3^/µL
Basophils	0	0.0-0.2×10^3^/µL
Coagulation panel		
Prothrombin time (PT)	15 seconds*	9-13 seconds
International normalized ratio (INR)	1.2	0.8-1.2
Activated partial thromboplastin time (APTT)	43.1 seconds*	25-35 seconds

The patient was started on treatment with cycle 1 of the R-HyperCVAD regimen (rituximab, cyclophosphamide, vincristine, doxorubicin, and dexamethasone) and initially achieved minimal residual disease (MRD)-negative remission as assessed by multiparametric flow cytometry (<0.01%) at the end of induction. He completed eight cycles of R-HyperCVAD and intrathecal chemotherapy with methotrexate and cytarabine (September 2022-May 2023). This was followed by POMP (6-mercaptopurine, Oncovin, methotrexate, and prednisone) maintenance therapy from June 2023 to February 2024.

**Figure 1:**
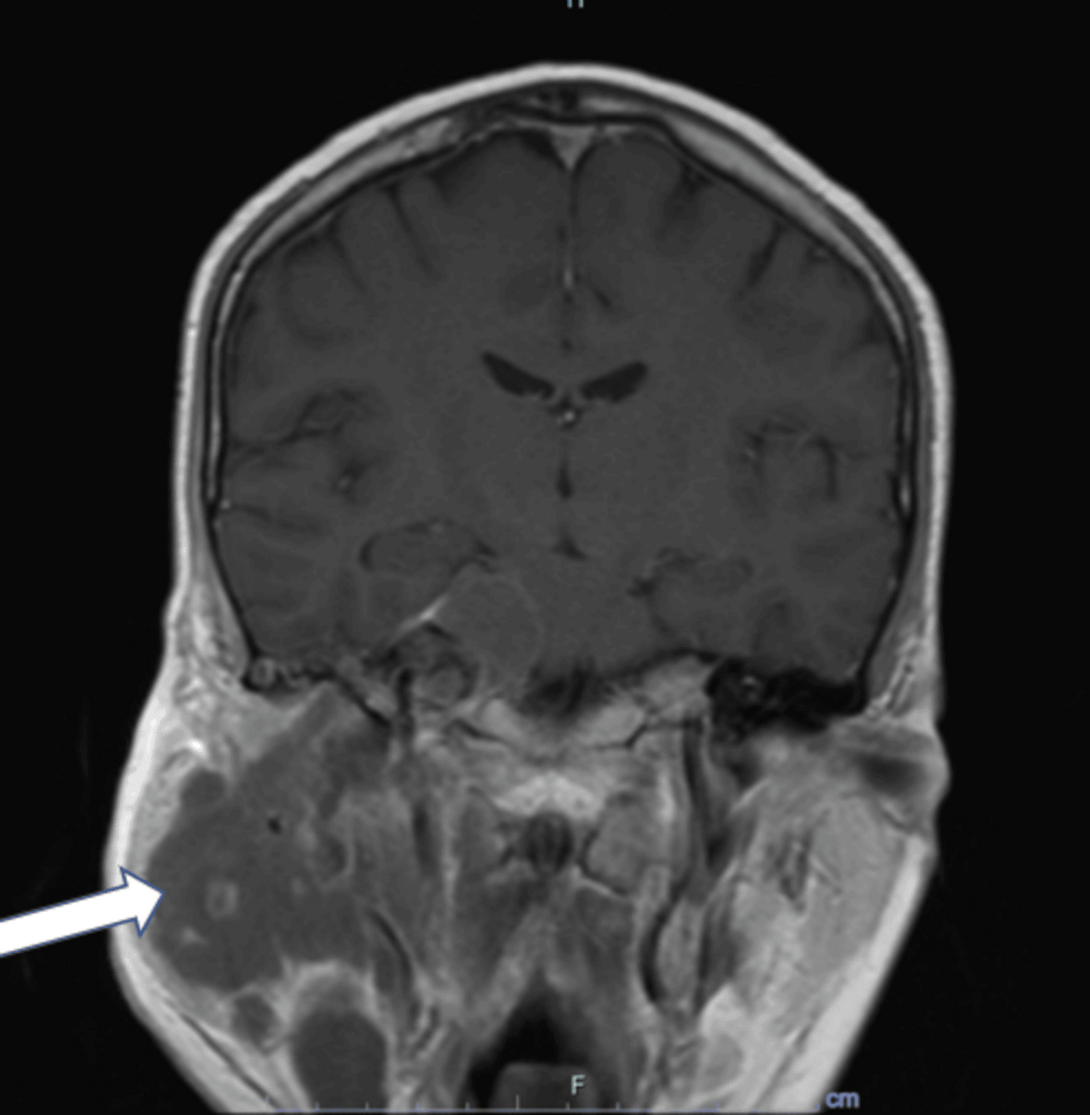
Post-contrast coronal T1-weighted MRI of the brain demonstrating a heterogeneously enhancing mass extending from the right cerebellopontine angle cistern to the right masticator space (white arrow), with extension along the extracranial and intracranial course of the right trigeminal nerve. It measures approximately 84×74 mm in maximal craniocaudal and transverse diameters. A parotid gland biopsy performed shortly after this imaging confirmed the relapse of B-ALL MRI: magnetic resonance imaging; B-ALL: B-cell acute lymphoblastic leukemia

In January 2024, the patient experienced a first relapse, presenting with right-sided facial symptoms. Brain magnetic resonance imaging (MRI) revealed an abnormality involving the intracranial and extracranial components of the trigeminal nerve and parotid gland with possible leukemic infiltration of Meckel's cave. Bone marrow biopsy and flow cytometry of the CSF were negative. In April 2024, a parotid gland biopsy was performed and confirmed the relapse of B-ALL (Figure 1). MRD positivity (4.7%) was demonstrated in the bone marrow, and atypical cells were identified on CSF flow cytometry. Salvage therapy, consisting of mini-CVD part B, was initiated, followed by sequential inotuzumab ozogamicin (InO) and blinatumomab. Brain imaging done six weeks after salvage chemotherapy demonstrated a new enhancing lesion in the left frontal lobe with a decrease in the size of the right trigeminal nerve mass (both intracranial and extracranial components) and a significant reduction in pachymeningeal thickening. MRD-negative complete remission was achieved in July 2024 after three cycles of salvage therapy with CSF negative for atypical cells.

**Figure 2:**
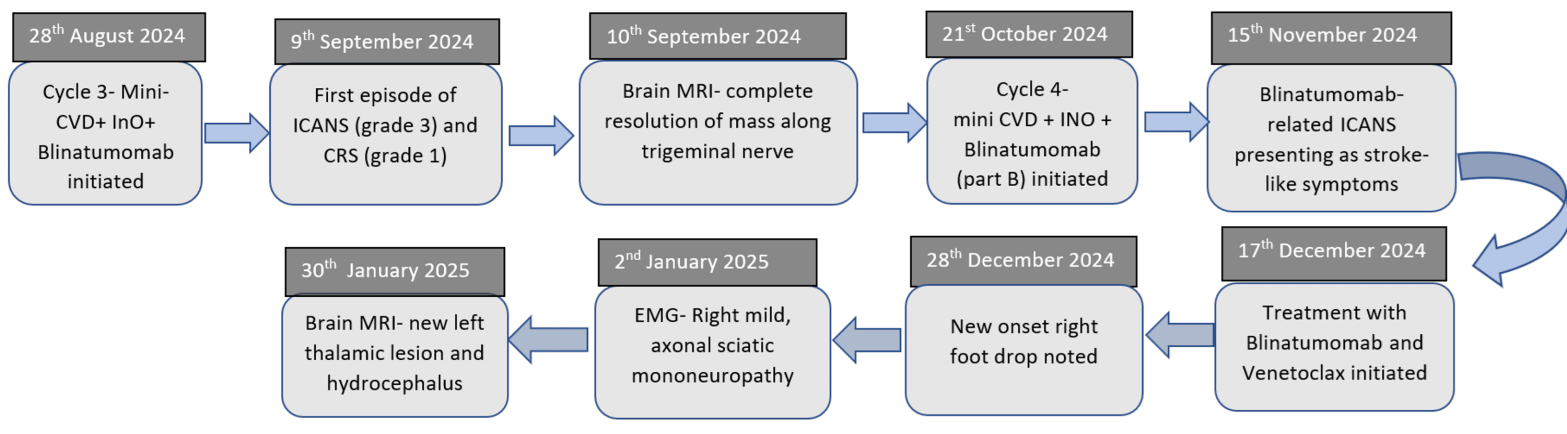
Chronological summary of key clinical events ICANS: immune effector cell-associated neurotoxicity syndrome; CRS: cytokine release syndrome; MRI: magnetic resonance imaging; EMG: electromyography

In November 2024, during the blinatumomab infusion, the patient developed acute-onset stroke-like symptoms presenting as facial and hand numbness and an inability to speak English, but his ability to speak Spanish (his native language) remained intact. This presentation was consistent with ICANS, following which the infusion was stopped and dexamethasone IV was administered. In December 2024, however, the patient developed right partial foot drop with bilateral lower extremity numbness. Neurology was consulted, and an electromyography (EMG) was done, which revealed right mild axonal sciatic mononeuropathy. The patient was started on treatment with intravenous immunoglobulin (IVIG) and steroids, with no significant improvement in the foot drop (Figure 2).

**Figure 3:**
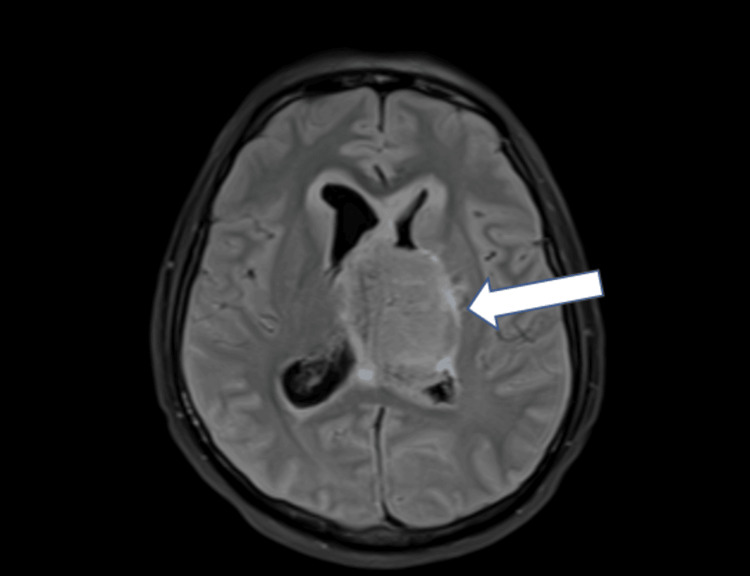
An axial image of brain MRI showing a large, well-defined lesion (white arrow) predominantly involving the third ventricle with compression of the lateral ventricles bilaterally and evidence of obstructive hydrocephalus. Adjacent brain parenchyma shows mass effect, including mild midline displacement MRI: magnetic resonance imaging

**Figure 4:**
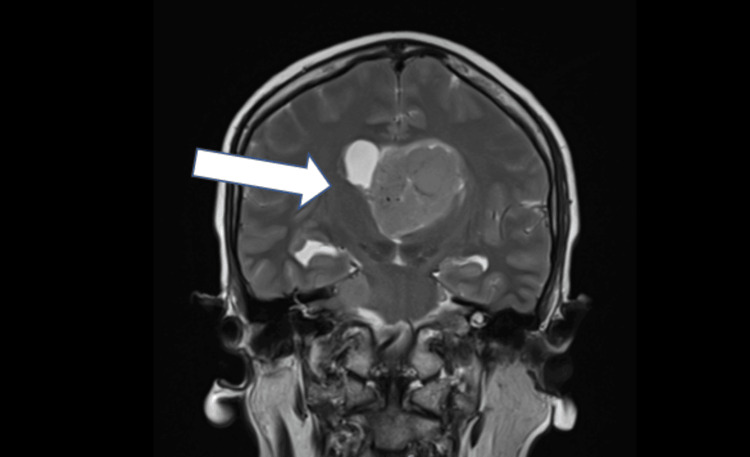
A coronal view of brain MRI showing a centrally located lesion within the third ventricle (white arrow) with a lobulated morphology and significant distortion of surrounding structures, including the compression of adjacent brain tissue and effacement of nearby cortical sulci MRI: magnetic resonance imaging

The patient presented with signs of a second CNS relapse, in January 2025, with right facial droop and numbness. Subsequent MRI of the brain revealed leptomeningeal disease progression and the development of hydrocephalus (Figures 3-4).

Over the course of his hospitalization, the patient became progressively drowsier and increasingly confused. He was transferred to hospice care, where he eventually passed away.

## Discussion

Blinatumomab, a bispecific CD19-directed CD3 T-cell engager, has been approved by the US Food and Drug Administration (FDA) since December 3, 2014, for the treatment of MRD-positive B-cell precursor ALL, relapsed or refractory B-cell precursor ALL, and B-cell precursor ALL in the consolidation phase in both adult and pediatric patients [7]. In the present case, blinatumomab was indicated for relapsed or refractory B-cell precursor ALL due to the detection of MRD at 4.7% in the marrow, which was detected with multiparametric flow cytometry. The patient received blinatumomab in combination with inotuzumab ozogamicin and achieved complete remission within three months. In a study conducted by Gao et al., which utilized data from the FDA Adverse Event Reporting System (FAERS) and included 5,962 patients, the median time to onset of blinatumomab-related nervous system toxicity was three days (interquartile range: 1-21 days) [8]. The symptoms observed in the present case have also been documented in this study, including ICANS (n=30; reporting odds ratio (ROR) 33.48; 95% confidence interval (CI) 23.34-48.03) and peripheral motor neuropathy (n=4; ROR 11.96; 95% CI 4.48-31.96) [8]. However, as the study is based on FAERS data, certain symptoms may have been categorized under broader preferred terms such as "neurotoxicity" or "neurological symptom" [8]. Based on a systematic review by Grant et al., the presentations of ICANS varied widely, with aphasia (88%) being the most common feature [9]. However, peripheral neuropathy was only mentioned in one of the 17 studies analyzed, without specifying the impaired region [9], and other discussed symptoms did not fit with the description of "foot drop" caused by sciatic axonal mononeuropathy. Furthermore, the incidence of neurological adverse events associated with blinatumomab has been shown to correlate with male sex and younger age (18-45 years) [8]. Another study also pointed out that the risk of ICANS is positively associated with an increased level of C-reactive protein, ferritin, and the presence and severity of CRS [9]. Moreover, traditionally, CNS involvement of leukemia was considered to be correlated with the frequency and severity of ICANS [10]. A post-hoc analysis by Leahy et al., utilizing data from five clinical trials with 195 patients involved, found that the incidence and severity of neurotoxicity did not significantly differ between the CNS-negative and CNS-positive disease strata (p=0.20) for any grade (53 (41%) vs. 38 (58%)), as well as for grade 1 (24 (19%) vs. 20 (30%)), grade 2 (14 (11%) vs. 10 (15%)), grade 3 (12 (9%) vs. 6 (9%)), and grade 4 (3 (2%) vs. 2 (3%)) [11].

Although ICANS is classically considered a CNS complication, there is growing recognition that the peripheral nervous system may also be affected in rare cases. The pathophysiology involves a cytokine-driven inflammatory cascade, disrupting the blood-brain and potentially the blood-nerve barrier. This disruption may facilitate infiltration of activated immune effector cells into peripheral nerves, leading to localized inflammation and axonal injury, particularly in vulnerable or anatomically constrained nerves such as the sciatic nerve. The resulting immune-mediated injury could manifest as a mononeuropathy, as seen in our patient [12]. 

While CAR-T-associated ICANS is similarly driven by cytokine release and endothelial activation, peripheral manifestations, including cranial neuropathies and Guillain-Barré-like syndromes, have been more frequently documented in CAR-T literature, supporting the plausibility of peripheral nerve involvement in other immune effector cell therapies such as blinatumomab [12,13].

Even with advancements in treatment, especially the introduction of rituximab, CNS relapse remains a significant challenge in B-ALL. Before rituximab became common, studies showed that 6-30% of adult B-ALL patients experienced CNS relapse [5]. These rates were influenced by factors like high-risk cytogenetic abnormalities such as the Philadelphia chromosome t(9;22), t(4;11), complex karyotypes, and hypodiploidy [14]. Additionally, high white blood cell counts, T-cell immunophenotype, and involvement of areas outside the bone marrow increased the risk of CNS involvement [14]. Intensive CNS-directed therapy, using methods such as prophylactic intrathecal chemotherapy and/or cranial irradiation, was important to lower this risk [15].

Rituximab, a monoclonal antibody that targets the CD20 protein on B cells, has transformed the treatment of many B-cell cancers [14]. It has improved outcomes when added to chemotherapy regimens for diseases like diffuse large B-cell lymphoma (DLBCL) [16,17]. As a result, rituximab was integrated into B-ALL treatment to reduce relapse rates, including CNS relapse. However, its actual impact on CNS relapse in B-ALL is still being studied. One major limitation is that rituximab does not penetrate the blood-brain barrier well. Studies have found that CSF concentrations of rituximab are only about 0.1% of serum concentrations after intravenous administration, which might not be enough to effectively eliminate leukemic cells in the CNS [18].

While rituximab may help control B-ALL systemically, its poor CNS penetration might not be enough to prevent CNS relapse, especially in patients with pre-existing CNS involvement or other high-risk characteristics. New strategies are needed to improve CNS penetration, like intrathecal rituximab [19], liposomal encapsulation of chemotherapeutic agents, or using newer drugs with better CNS availability. Further research into how B-ALL spreads to the CNS and the development of targeted therapies is crucial to improve outcomes for these patients.

## Conclusions

This case highlights the complexities of managing relapsed/refractory B-ALL with CNS involvement, particularly in the setting of immunotherapy with agents like blinatumomab. Despite achieving MRD-negative remission, the patient experienced multiple CNS relapses, emphasizing the persistent challenge of preventing leukemic infiltration in sanctuary sites. The development of ICANS-associated foot drop further illustrates the spectrum of blinatumomab-induced neurotoxicity, which may affect both central and peripheral nervous systems. These findings underscore the importance of close neurological monitoring and the need for enhanced CNS-directed strategies. Given the expanding role of immunotherapies, incorporating targeted neurodiagnostic tools such as MRI and EMG may facilitate earlier distinction between immune-mediated neurotoxicity and disease progression. Future research should aim to improve CNS drug delivery and optimize treatment protocols that balance therapeutic efficacy with neurotoxicity risk, particularly in high-risk patients.
